# A Population Genetic Model for the Initial Spread of Partially Resistant Malaria Parasites under Anti-Malarial Combination Therapy and Weak Intrahost Competition

**DOI:** 10.1371/journal.pone.0101601

**Published:** 2014-07-09

**Authors:** Yuseob Kim, Ananias A. Escalante, Kristan A. Schneider

**Affiliations:** 1 Department of Life Science and Division of EcoScience, Ewha Womans University, Seoul, South Korea; 2 School of Life Sciences and Center for Evolutionary Medicine and Informatics at the Biodesign Institute, Arizona State University, Tempe, Arizona, United States of America; 3 Department of Mathematics, University of Applied Sciences, Mittweida, Germany; Johns Hopkins Bloomberg School of Public Health, United States of America

## Abstract

To develop public-health policies that extend the lifespan of affordable anti-malarial drugs as effective treatment options, it is necessary to understand the evolutionary processes leading to the origin and spread of mutations conferring drug resistance in malarial parasites. We built a population-genetic model for the emergence of resistance under combination drug therapy. Reproductive cycles of parasites are specified by their absolute fitness determined by clinical parameters, thus coupling the evolutionary-genetic with population-dynamic processes. Initial mutations confer only partial drug-resistance. Therefore, mutant parasites rarely survive combination therapy and within-host competition is very weak among parasites. The model focuses on the early phase of such unsuccessful recurrent mutations. This ends in the rare event of mutants enriching in an infected individual from which the successful spread of resistance over the entire population is initiated. By computer simulations, the waiting time until the establishment of resistant parasites is analysed. Resistance spreads quickly following the first appearance of a host infected predominantly by mutant parasites. This occurs either through a rare transmission of a resistant parasite to an uninfected host or through a rare failure of drugs in removing “transient” mutant alleles. The emergence of resistance is delayed with lower mutation rate, earlier treatment, higher metabolic cost of resistance, longer duration of high drug dose, and higher drug efficacy causing a stronger reduction in the sensitive and resistant parasites’ fitnesses. Overall, contrary to other studies’ proposition, the current model based on absolute fitness suggests that aggressive drug treatment delays the emergence of drug resistance.

## Introduction

Effective antimalarial drugs remain a major means of controlling human malarias. However, control efforts of *Plasmodium falciparum*, the most virulent form of human malaria, have been thwarted by the rapid evolution of drug resistance. Indeed, after about 12 years of massive use of chloroquine (CQ), mutations conferring resistance against CQ emerged independently in four geographic regions [1]–[3]. In the 1990s, CQ became ineffective to treat *P. falciparum* malaria in many endemic areas worldwide. It was replaced as a first-line treatment by sulphadoxine-pyrimethamine (SP), a combination of two drugs. SP resistance emerged quickly and spread across endemic areas [4]–[6]. Currently, arteminisin-based combination therapies (ACT) have become the preferred treatment option. The rationale behind combination therapies is that a parasite acquires resistance only when it carries independent mutations, each of which acts against a single drug [1], [7],[8]. However, the recent observation of weak ACT resistance in Southeast Asia [9]–[14] may forecast further evolution of clinical resistance that threatens successful control interventions and highlights the importance of understanding the mechanisms driving drug resistance under combination therapy.

Theoretical investigations have followed two paths: one focusing on a characterization of the patterns emerging from empirical data (e.g. [15]), the other directed to understanding how rapidly drug resistance occurs and what biological/clinical factors affect its spread. The latter path was mainly approached by either models of population/demographic dynamics or by population/evolutionary genetic processes. Although there is a general agreement on the emerging patterns [16], they differ in several key elements. Population/demographic models focus on demographic dynamics of infected *vs.* uninfected hosts, as determined by epidemiological factors such as transmission patterns of sensitive and resistant parasites, drug treatment, and immunity [17], [18]. This approach is useful to understand the circumstances allowing or suppressing the spread of resistant parasites; however, usually at the cost of ignoring the evolutionary dynamics of resistant mutations. On the contrary, population/evolutionary genetic models reduce this problem to the temporal change of a resistant allele’s frequency in the pathogen population [1], [15],[16],[19]–[25]. Mathematical models here often assume a homogeneous pathogen population of constant or infinite size, ignoring the complex demographic structure of parasites that is naturally shaped in endemic regions.

Regardless of their limitations, population-genetic models allow the description of the early phase of drug-resistance evolution – a sensitive allele randomly mutates into a resistant allele and increases in frequency. In contrast, population-dynamic approaches usually assume that mutations conferring resistance are already in relative high frequency in the population. Drug selection is the driving force of their dynamics and they neglect the early phase when resistance mutations can be easily lost by random drift. However, a crucial problem in public-health planning of drug deployment is to predict the number of *de novo* resistant mutations arising in the pathogen population upon drug treatment and the likelihood that they survive stochastic loss and swamp the entire population. Such information is essential for monitoring/containment of resistance at early stages. This prediction can only be made from accurate modelling of the early population-genetic processes [26]. Another reason to use population-genetic modelling is that disease dynamics may be critically dependent on the genetic details of resistance. For example, molecular genetic analyses of major genes involved in anti-malarial drug resistance showed that multiple non-synonymous mutations occurred at each responsible gene and their order of occurrence determines the degree of resistance [27] and therefore translates into difference in fitness [5]. Such genetic details may not be properly addressed by population-dynamic approaches.

Despite these advantages, there still remains serious limitations regarding population dynamics in population genetic approaches as mentioned above. Conventional population-genetic models are based on the discrete-time Wright-Fisher model or its variants [28], which describe the process of inheritance as random sampling of gametes for a fixed number of individuals in a given generation. Here, a simple assumption (or no explicit assumption) about population size or population structure is made because it needs to be specified as a parameter for the sampling. Then, the entire system is described by the temporal change of relative frequencies, not absolute counts, of alleles under consideration. The most important determinant of the model is therefore the relative fitness of different alleles, which determines the trajectory of relative allele frequencies. However, in a realistic epidemiological model, the size and structure of pathogen populations under density dependence should be a key variable rather than a fixed parameter. For example, a given host shows clinical symptoms only if parasitaemia (parasite density inside the host) exceeds a threshold value [29], which is followed by drug treatment that subsequently reduces parasitaemia and also changes the fitness landscape of different alleles. Therefore, there is an intimate feedback between demographic and genetic processes during drug-resistance evolution - at least at the level of the parasite populations inside human hosts.

Currently, two contrasting recommendations exist for drug-deployment policies that aim to maximize the life span of anti-malarial drugs by delaying the evolution of resistance, interestingly, both rooted in the principles of population genetics. First, strong and thorough drug treatment that ensures complete clearance of parasites is recommended [30], [31] to minimize the sensitive parasites’ probability to mutate to resistant ones. This is understood given the principle that the rate of adaptation by natural selection increases with increasing population size, which leads to an increasing number of new mutations entering the population and increasing efficacy of selection [32]. Therefore, one might expect that keeping the number of sensitive parasites as low as possible by aggressive drug treatment would delay the emergence of resistance. In disagreement, it is argued that stronger drug treatment only increases the relative fitness of resistant mutants, thus accelerating their spread [33], [34]. This argument is particularly true in the presence of strong intrahost competition between sensitive and resistant parasites, which was experimentally demonstrated in mice [35]. Therefore, contrary to the first, the second recommendation suggests to minimize drug usage to avoid creating a fitness landscape favourable for evolving drug resistance.

It might be too simplistic to suggest adopting only aggressive or minimal drug as a general and exclusive principle. It was argued that aggressive chemotherapy would be effective in deterring the rise of resistance starting from *de novo* mutation but facilitate its spread later when a strongly resistant allele has already reached a sufficient frequency in the population [34]. However, at which point (in the timeline of resistance evolution) treatment options should be switched was not made clear. Whether and where such a switch occurs will critically depend on how effectively strong drug treatment eliminates both sensitive wild-type and resistant mutant parasites, while the mutant has higher fitness relative to the wild-type under the initial dose of drugs. Conventional population-genetic modelling based only on relative fitness cannot describe such a scenario and thus cannot evaluate its importance in the overall evolutionary outcome. It appears that resolving these conflicting conclusions requires the quantitative assessment of a more complete model. Such a model must consider the absolute as well as relative numbers of resistant parasites at both intra- and interhost levels. Importantly, it needs to consider the whole evolutionary process starting from *de novo* mutations in sensitive parasites, which rise to high frequency among parasites in mosquitoes. This consideration again highlights the importance of a model that integrates demographic and genetic dynamics.

In this study, a stochastic model of anti-malarial drug-resistance evolution is proposed. The major aim is not to provide accurate quantitative predictions regarding the speed of resistance evolution but to demonstrate that drug resistance can arise and spread in a manner that was not considered before if both population-dynamic and genetic processes are effectively integrated in the model. A population of malaria parasites is considered and their reproduction is modelled in terms of absolute fitness (expected number of offspring in the next generation), rather than relative fitness. The absolute fitness is mainly specified by a function of drug usage in the respective host. Then, the behaviour of the proposed system is examined using numerical simulation. The model does not intend to recreate all aspects of genetics and population dynamics associated with resistance evolution. However, it is designed to address the following fundamental properties of resistance evolution in sufficient detail. First, two loci associated with resistance against two different drugs are considered, as the current paradigm of drug treatment against pathogenic parasites is to simultaneously administer two or more different drugs (e.g. SP or ACT). Second, the simulation model strives to be accurate with regard to early dynamics capturing the period in which drug treatment starts in a host population but a resistant mutation has not yet started to propagate. Notably, genetic analyses revealed that both CQ and SP resistance spread from surprisingly few mutational origins [18], [27], [36] followed by widespread geographical migration. This implies that the propagation of resistant alleles does not occur immediately after the introduction of drugs and a realistic model should yield a reasonably long waiting time before resistance spreads. Dissecting compounding factors that govern the waiting time until such a rare occurrence is a major objective of this model. Third, in line with the above objective, only partially resistant mutations are considered. Namely, while partially resistant parasites are cleared out less efficiently by the drug than sensitive ones, a sufficient dose of drugs still kills them [37], [38]. Thus, the current model focuses on the initial spread and establishment of mutations conferring partial resistance that is typically manifested by single initial amino-acid mutants (weakly resistant allele). This might be the most important step in determining the entire waiting time for the emergence of full drug resistance. Finally, it is aimed to build a flexible and computationally feasible numerical model of resistance evolution which is extendable to further demographic and genetic details.

## Methods

### Model outline

**Figure 1:**
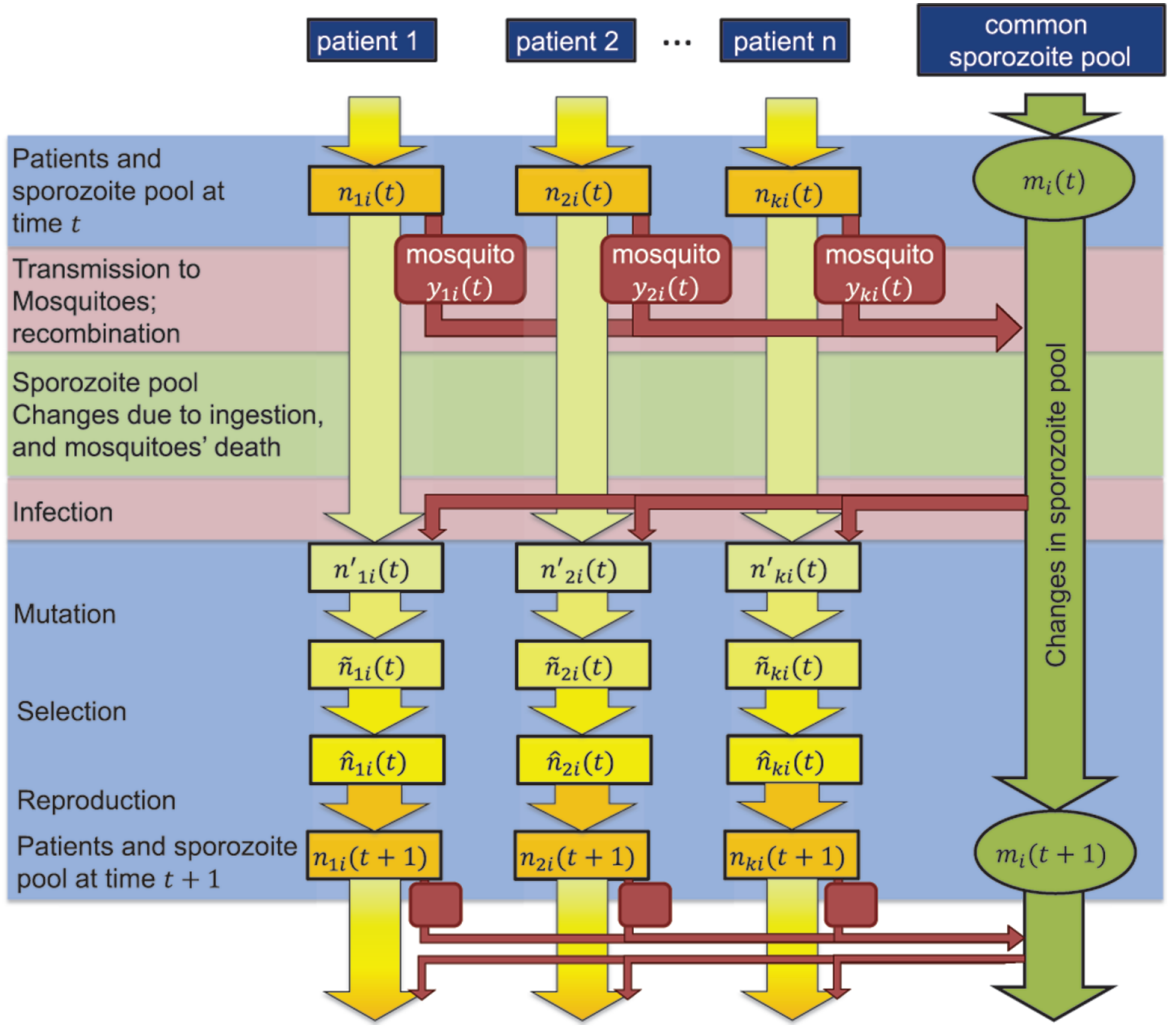
Schematic diagram of parasite reproduction cycle in the model. In discrete time unit, the numbers of parasites in hosts and in the mosquito infective pool are updated.

The temporal changes in the numbers of sensitive and resistant parasites transmitted among a finite number of human hosts that are susceptible to infection are of primary interest. The dynamics of the system is modelled in discrete time as described below (see Figure 1 for an illustration of the dynamics from time *t* to time *t*+1). For modelling purposes, parasites within a host are treated as a sub-population of the total population of all parasites, analogous to Wright’s island model [28]. Moreover, there is an additional sub-population termed migrant pool or mosquito pool, which reflects the collection of parasites that are transmitted among all mosquitoes. Hence, human hosts are modelled explicitly but mosquitoes only collectively. The dynamics of parasites within a host determines the course of the infection and its treatment. If the number of parasites within a host exceeds a critical value (*N*_C_), the infection is “detectable” (i.e. the patient expresses disease symptom) and the host receives drug treatment. Thus, the parasitaemia threshold, *N*_C_, represents how early infections are treated. A treated host receives a combination of two drugs, D_1_ and D_2_, which clear out parasites. If the cumulative parasitaemia in a host during the period of consecutive infection, despite drug treatment, exceeds another threshold, the infection is terminated (e.g. the patient dies or is cured by another effective drug instantaneously). Within the host, haploid parasites reproduce by mitosis and might mutate to confer partial resistance against the drugs. During the course of the infection, a small number of parasites enter the migrant pool, as mosquitoes take their blood meal from infected hosts. Random pairs of parasites that exit from the same host conjugate and recombine in the migrant pool. This models gametocytes of *Plasmodium* species recombining immediately after the blood meal within the mosquitoes’ guts, and mature into sporozoites that reside in the mosquitoes’ salivary glands. All haploid parasites (sporozoites) within the mosquitoes are treated as if they constitute a single deme, which sends migrants back to hosts. It models parasites’ migration between hosts via mosquito vectors in which recombination occurs. Although, malaria parasites undergo many developmental changes, i.e., sporozoites, merozoites, gametocytes, etc., for modelling purpose this fine distinction is not needed. Therefore, all these forms are simplistically subsumed just as parasites. This is a reasonable assumption if the genetic compositions of parasites in the respective stages are proportional.

### Genetic assumptions

Parasites within the host are haploid and reproduce asexually, whereas they undergo a step of meiosis within mosquitoes when they migrate between hosts. There are two loci, A and B, in the parasite’s genome that are associated with (partial) resistance against drug D_1_ and D_2_, respectively. Each locus has two alleles, a sensitive (*A_S_* or *B_S_*) and a resistant (*A_R_* or *B_R_*) one. Therefore, each parasite has one of the four haplotypes *A_S_B_S_*, *A_S_B_R_*, *A_R_B_S_*, and *A_R_B_R_*, indexed as haplotype 1 (“sensitive”), 2 and 3 (“single-drug resistant”), and 4 (“double-drug resistant”), respectively. Their life cycle includes a brief diploid phase that ends with meiosis. It is assumed that recombination between the loci occurs during meiosis with probability *r*. The growth rate of parasites (absolute fitness) within hosts depends on their haplotype, i.e., whether they are sensitive or resistant against drugs. Moreover, sensitive parasites within hosts might mutate to confer resistance. Summarizing, selection and mutation occur within hosts, whereas recombination occurs outside the hosts in the mosquito vector.

It is assumed that the population of parasites evolves in discrete time, which is simply counted as “generations”. One generation does not necessarily reflect the time span of an infection, or a full transmission cycle, although it was originally intended to roughly correspond to 12 hours for the following two reasons. First, because drugs clear parasites continuously, a finer time scale than the usual 48-hour cycle seems appropriate. Second, to properly model transmission it was important to choose a generation time reflecting that mosquitoes can bite anytime. However, because other time-dependent parameters described below were chosen arbitrarily, this time unit can essentially be scaled arbitrarily.

### Drug pressure within human hosts (selection)

In total, there are *H* potential hosts (corresponding to demes in a migration framework). The parasite population is divided into subpopulations corresponding to hosts they reside in. In host *j* ( = 1, …, *H*), the number of parasites with haplotype *i* at time *t* is denoted by *n_ij_*(*t*). The change of parasite numbers within a host depends on the parasites’ haplotypes, the total number of parasites (density) in the host and drug concentrations. The absolute fitness (the expected number of descendants in the next generation) of an individual parasite with haplotype *i* in host *j* is given by
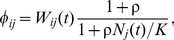
(1)where 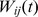 is the density-independent relative fitness of haplotype *i* in host *j* at time *t*. 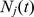 is the total number of parasites (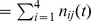) in host *j*, ρ is the intrinsic growth rate (parasite number multiplies by 1+ρ at low density if 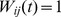) and *K* is the carrying capacity of parasites in an untreated host. Assume that the concentrations of drug D_1_ and D_2_ in host *j* are 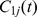 and 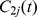, respectively, at time *t*. Naturally, 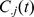 is normalized to 0 in the absence of the drug and 1 at the maximum therapeutic concentration (the initial dose). Then, 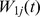 describes the impact of drugs on the fitness of sensitive parasites under the assumption that fitness is correlated with drug concentrations:



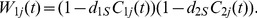
(2a)The parameter *d_kS_* therefore represents the effect of drug *k* on the sensitive parasite. 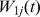 decreases from 1 to 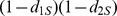 as the concentrations of both drugs increase. Next, we define
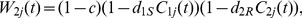
(2b)

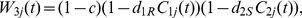
(2c)

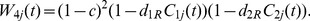
(2d)


In the above equations, *c* represents the strength of natural selection against the resistant allele (“metabolic cost of resistance”) and *d_kR_* represents the effect of drug *k* on the resistant parasite. Drug resistance means *d_kS_*>*d_kR_*≥0. Drug concentrations, 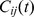, will be given as functions of time according to the specific model of drug treatment described below. Note that, since eq. (1) is a decreasing function of *N_j_*(*t*), the fitness of a resistant parasite increases as the number of sensitive parasites in the same host decreases. However, as long as 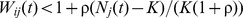, its absolute fitness remains below one. Namely, sufficiently strong drugs do not allow partially resistant parasites to grow when sensitive parasites were cleared, contrary to other models that assume strong within-host competition [33], [34].

### Reproductive cycle

Migrations of parasites between hosts, corresponding to new infections and co-infections, occur in each generation. No spatial structure is assumed in the host population. Therefore, hosts receive infective parasites (“migrants” in the context of the Wright’s island model) equally from a common mosquito pool (migrant pool), which models the collection of parasites in mosquitoes’ salivary glands. Let 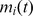 be the expected number of parasites (sporozoites) of haplotype *i* at time *t* that reside in the mosquito pool that are ready to be inoculated into hosts. After, a mosquito took its blood meal; parasites (gametocytes) undergo a step of meiosis before new parasites (sporozoites) are developed. Hence, 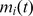 does not change solely due to transmission from all hosts to mosquitoes, but also undergo a step of sexual reproduction. In particular, changes occur as follows:

The number of parasites contributed by a given host to the migrant pool depends on its current load of parasites. Since the number of parasites (gametocytes) transferred from host *j* to a mosquito increases with *N_j_*(*t*) but not linearly [39], [40], we model that the contribution of host *j* to the migrant pool is proportional to log(1+*N_j_*(*t*)). The haplotype frequencies of migrants (infective sporozoites) originating from a host are different from those in that host because meiotic recombination between haploids occurs within the mosquito (see Figure 1). Let *y_ij_* be the expected frequency of haplotype *i* descending from host *j*. Then, using the standard recursions for haplotype changes by recombination [28],
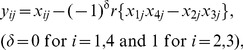
(3)where *r* is the recombination rate between the two loci and 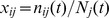. Therefore, the number of sporozoites in the migrant pool changes according to
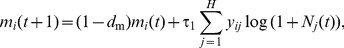
(4)where τ_1_ specifies the rate of parasite migration (transmission) from host to mosquito and *d*_m_ is the death rate of mosquitoes.

The recursive changes of 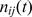 are given by the following equations. Due to parasite migration (infection), the number of parasites in a host increases to
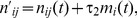
(5)where τ_2_ specifies the rate of infection/transmission from mosquitoes to hosts. It is important to notice that τ_1_ (equation 4) and τ_2_ summarize the vector population density and/or vector competence. Note that the losses of parasite numbers as gametocytes out of hosts and as sporozoites out of the mosquito pool are ignored. Furthermore, within hosts, mutation happens at each locus with probability µ per generation. Simultaneous mutations at both loci are ignored, since they are very unlikely. Thus, the expected numbers change to 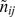, where



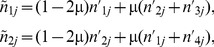
(6a-b)

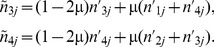
(6c-d)


At time *t*, transmission between mosquitoes and hosts occurs. Simultaneously, hosts are co-infected from the common sporozoite pool. Next, mutation and replication (selection) occur. After replication the expected numbers of haploids become
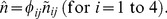
(7)


Finally, in order to account for the stochastic dynamics of parasite reproduction, the number of parasites with haplotype *i* in host *j* in the next generation is assumed to be determined by
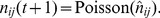
(8)


Of course all steps in the recursion, particularly migration (transmission) in and out of hosts, are of stochastic nature. However, it is assumed that the above Poisson distribution absorbs the stochastic noises generated in those steps. We choose a Poisson distribution because it approximates the offspring number distribution under the Wright-Fisher model of reproduction. However, unlike the Wright-Fisher model, population size is not a pre-determined parameter but a dependent variable that changes at a rate determined by the genetic composition of population, the change of which is in turn modulated by within-host population size (i.e. increase above *N*_C_ triggering change in haplotype fitness). Therefore, the temporal dynamics of demography is now tightly coupled with that of the population’s genetic composition.

### Models of drug treatments and termination of infection

An infection is treated if the parasitaemia (number of parasites within the infected host) exceeds a given threshold value *N*_C_. We initially investigate the scenario in which all infected hosts are detected and treated. However, as even infections with high parasitaemia might be asymptomatic and hence untreated, we later allow only a fraction α of hosts to receive treatment by setting the carrying capacity of the remaining (fraction 1-α) hosts to be 0.1 *N*_C_. An infection is treated with a combination of two drugs, which will lead to rapid clearance of parasites within the hosts. More specifically, immediately after the total number of parasites in the host exceeds the critical value, *N*_C_, drugs are administered. It is further assumed that the concentration of the drugs remains constant for *l*_T_ generations after initial administration and then decays exponentially. Let 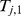 be the first time the host *j* takes drug *i*. Subsequently, 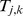 denotes the time of the *k*th drug administration.

Then, the concentration of drug *i* ( = 1 or 2) in host *j* at time *t* is given by
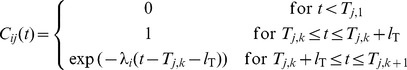
(9)Here, λ*_i_* specifies how fast the concentration of drug *i* decays. Clearly, we have, 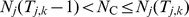, where 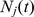 is the total number of parasites in host *j* at time *t* before selection.

If a host is infected with a large number of resistant parasites, drug treatments do not lead to clearance. Under the model formulated above, such a host is subject to repeated cycles of treatment and recrudescence. While prolonged infection with resistant parasites without clinical manifestation often occurs in reality, we may also consider the termination of resistant infection due to host death or treatment with an alternative effective drug. We assume that, if the cumulative parasitaemia starting from a new infection exceeds *k*_D_ *N*_C_ in a host, parasite numbers in that host is set to zero (*n_ij_*(*t*) = 0 for all *i*). Increasing *k*_D_ from small to large value thus allows effectively modelling a range of scenarios, from host death with replacement or radical cure of the infection with an alternative effective drug to recrudescence without clinical manifestations.

### Simulation

The numerical simulation starts with 100 wild-type (sensitive) haploids (sporozoites) in the mosquito pool that are ready to be transmitted to hosts (i.e. *m*_1_(0) = 100, and *m_i_*(0) = 0 for *i*≠1). Namely, it simulates the scenario that malaria parasites are introduced to a host population carried by a small number of infected mosquitoes. (However, as shall be seen in the results, an endemic state will be reached before the spread of resistant parasites is successfully initiated.) If parasites are lost from the population by chance (*n_ij_*(*t*) and *m_i_*(*t*) = 0 for all *i*, *j*) the simulation starts again. One replicate of a simulation is run up to *T*_max_ generations and the frequencies of resistant alleles at both loci in the migrant pool are recorded. After the drug treatment starts (*i.e.* parasitaemia exceeds *N*_C_ in at least one host), there are three possible outcomes: 1) the resistant allele(s) at one locus (or both loci) exceeds a high frequency (0.5 in the mosquito pool; see below) before *T*_max_; 2) resistant alleles remain at low frequency until *T*_max_; or 3) all parasites, either sensitive or resistant, are eliminated before *T*_max_. If the first outcome is obtained in many simulation replicates (at least 100 runs) for a given parameter set, the mean waiting time is recorded. The entire simulation was written in C, which is available upon request. The stochastic effect is simulated by random Poisson number generator in (Press *et al.* 1992). The C code was validated by implementing the program in MATLAB (version R2009b; The MathWorks, Inc.) and comparing results.

## Results and Analysis

The numerical simulation of drug resistance evolution under combination therapy was conducted as described in Methods and examined how rapidly drug resistance alleles reach high frequency. Parameter values for the simulations (Table 1) were chosen so that a wide exploration of the parameter space would not become computationally too demanding. In particular, the population of 1,000 human hosts was used. To facilitate the emergence of drug resistance in such an unrealistically small host population, a high mutation rate was used. Other parameters were also chosen arbitrarily and may deviate considerably from the actual values in malaria epidemics. However, the major objective here is to identify evolutionary pathways leading to the rise of resistance and factors qualitatively influencing these pathways It is to be examined if change in absolute values of parameters lead to qualitative changes in the dynamics.

**Table 1:** Parameters used in the current model.

symbol	Definition*	starting value
*K*	Carrying capacity of sensitive parasites in an untreated host	10^11^
ρ	Intrinsic growth rate	1.5
*d_m_*	Death rate of mosquitoes	0.1
τ_1_	Transmission rate from host to mosquito	0.015
τ_2_	Transmission rate from mosquito to host	10^−5^
*r*	Recombination rate	0.5
µ	Mutation rate	10^−7^
*H*	Total number of hosts	1000
*N*_C_	Threshold number of parasites in a host that triggers drug treatment	5×10^10^
*k*_D_	Multiplying factor for the cumulative number of parasites ( = *k*_D_ *N*_C_) that causes host death	10
λ_1_ (λ_2_)	Rate of decay for drug 1 (drug 2)	0.1
*l*_T_	Duration of maximal drug concentration sustained after administration	5
*d*_1*S*_ (*d*_2*S*_)	The reduction of sensitive parasite’s fitness by drug 1 (drug 2)	0.95
*d*_1*R*_ (*d*_2*R*_)	The reduction of resistant parasite’s fitness by drug 1 (drug 2)	0.6
*c*	Selective disadvantage of a resistant allele in an untreated host (metabolic cost)	0.1
*T*_max_	Maximum number of generations for one simulation replicate	2×10^5^

*all time-dependent parameters are given per generation or in units of generations.

### Exemplary trajectories to drug resistance

**Figure 2:**
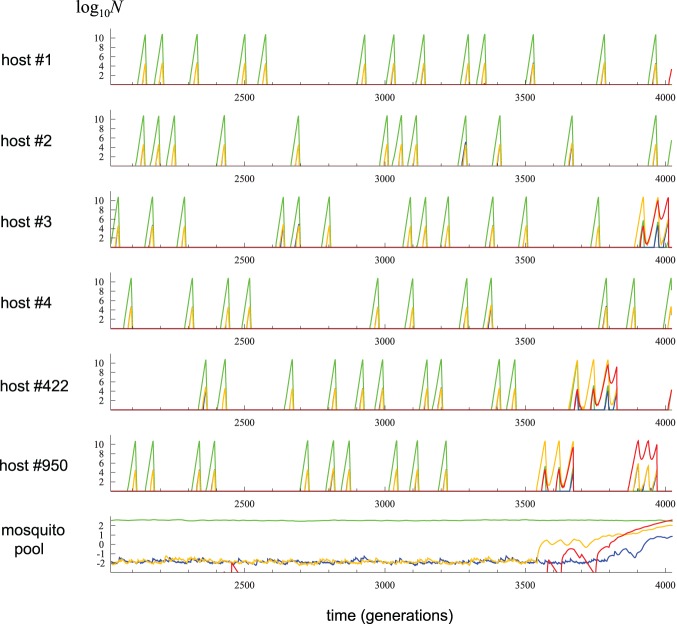
A representative replicate of the stochastic simulation, leading to the spread of drug-resistant parasites through type A establishment, run with parameters given in **Table 1**. The numbers of parasites with different haplotypes (1, 2, 3, and 4 by green, orange, blue, and red curves, respectively) are plotted over time for four randomly chosen hosts (#1–4) and for two hosts (#950, #422) where the establishments of single- and double-drug resistant strains, respectively, occur. Note that, as haplotype 2 and 3 are observed in similar numbers before the establishments, blue curves appear invisible behind orange curves. The parasite numbers were taken immediately after Poisson sampling (eq. 8; therefore non-negative integers) and their log_10_- transformed values are shown on the y-axis. On the bottom is the corresponding numbers of parasites (“migrants”) in the mosquito pool (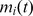; eq. 4). Note that 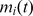 is the expected number (real-valued) of parasites and therefore can be a very small number.

The first step in understanding the evolutionary dynamics leading to the emergence of drug resistance (high frequency of *A_R_* and/or *B_R_*) under the proposed model is to identify key demographic and genetic changes that initiate the spread of resistant alleles. For this purpose the trajectories of parasite numbers with different haplotypes, from a randomly chosen replicate of the stochastic simulation, are plotted and closely inspected (Figure 2). The intrahost concentrations of the two drugs are assumed to decay at the same rate. All parameter values are shown in Table 1 (in the column “starting values”). Figure 2 shows the trajectories of parasite numbers in the mosquito migrant pool and in six hosts, four of which (host number 1 to 4) were arbitrarily chosen to show the dynamics at ‘average’ hosts. Host no. 950 is the first in which single-drug resistant parasites (*A_R_B_S_* in this example, shown by the orange curve) dominate over sensitive parasites (satisfying the condition that 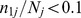 and 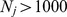). Host no. 422 is the first, in which double-drug resistant parasites (*A_R_B_R_*, shown by red curves) dominate over all other haplotypes (satisfying the condition that 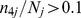 and 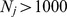). The entire course of simulation can be divided into two phases. Phase I is from the start of the simulation to the “establishment” of single-drug resistant parasites in host no. 950 in generation 3550. Phase II is from the end of phase I to the end of the simulation run when the relative frequency of double-drug resistant parasites (haplotype *A_R_B_R_*) exceeds 0.5 in the migrant pool.

During phase I, recurrent infections by sensitive parasites (shown by peaks of green curves) occur in all hosts. In each infection the parasite number exponentially increases to *N_C_* and triggers drug treatment, by which the number of sensitive parasites is quickly reduced to zero. Small numbers of single-drug resistant parasites (*A_R_B_S_* and *A_S_B_R_* shown by orange and blue curves, respectively) are generated by mutations when sensitive parasites are increasing to a large number. At the time of drug treatment in a host *j*, 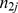 and 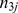 reach peaks that are greater than the single-generation production of new mutants (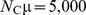), because mutations have also accumulated from previous generations, but obviously smaller than the expectation under the assumption of mutation-selection balance and constant population size at *N*_C_ (because each resistant allele lasts about 1/*c* generations, the expectation is 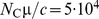). Combination therapy almost always wipes out these single-drug resistant parasites (but see below). Therefore, by tracking the haplotype dynamics in individual hosts a preliminary conclusion is drawn: higher relative fitness of resistant over sensitive parasites in drug-treated hosts does not result in immediate rise of resistant parasites despite repeated cycles of drug treatments. Effective combination drug treatment thus seems to suppress the spread of resistant alleles, as indicated by very low values of 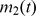 and 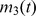 in the migrant pool, during phase I.

**Figure 3:**
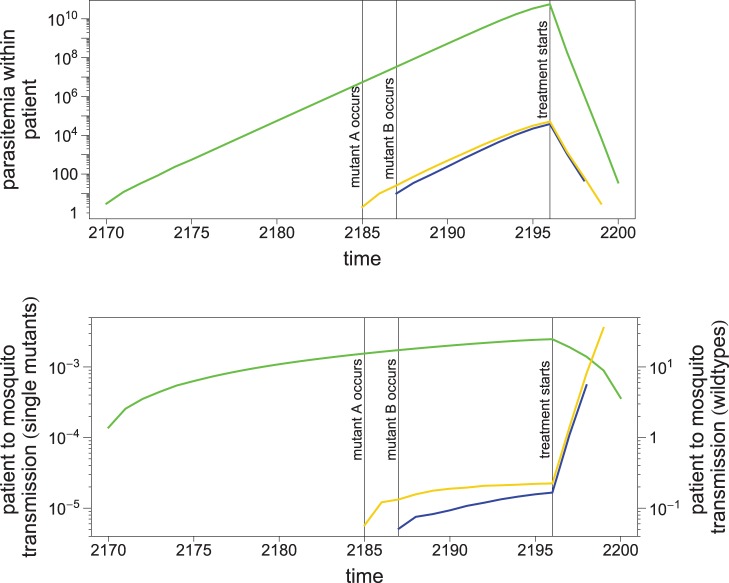
The transmission of parasites from a host to mosquitoes during the course of infection and clearance by drugs in phase I. Upper panel shows exemplary parasite numbers taken from simulation. Lower panel shows the corresponding temporal profile in the relative amount of host-to-mosquito transmission (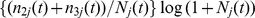; see eq. 4) for each haplotype (1, 2, and 3 shown in green, orange and blue, respectively). Note different y-axis scales for haplotype 1 and haplotype 2/3.

During the short-lived periods of presence in hosts, some single-drug resistant mutants may be transferred to the migrant pool. This, however, occurs with a very small probability and 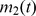 and 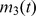 fluctuate at very low values during phase I. It should be noted that the transmission of a resistant parasite from a host to the migrant pool is most likely to happen shortly after drug treatment, when both sensitive and single-drug resistant parasites are being eliminated. This is because the contribution of host *j* to the migrant pool is modelled to be proportional to 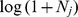 (eq. 4). Since single-drug resistant parasites are eliminated slowly relative to sensitive ones, their relative frequency, 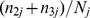, within host *j* increases sharply before 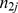 and 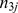 hit zero. The expected number of single-drug resistant mutants transferred from host *j* is proportional to 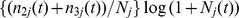 (eq. 4). Figure 3 shows that this quantity greatly increases upon drug treatment. Therefore, although the absolute fitness of resistant haplotypes under drug treatment is not high enough to start the immediate spread of resistance, the higher *relative* fitness of them leads to a considerable enrichment of resistant parasites in the mosquito pool. (This enrichment is seen in Figure 2 as 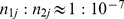 on average among hosts and 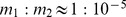 in the migrant pool during phase I).

Phase II starts when a host (no. 950 in Figure 2) is infected by a parasite with single-drug resistant haplotype (haplotype 2 in the example), due to a rare event of transmission from the mosquito pool. Obviously, drug concentration in this host was effectively zero at the time of infection, as the last drug treatment occurred about 300 generations ago while concentration decays by approximately 10% per generation (λ = 0.1), allowing the growth of this partial resistant parasite. The expected waiting time until such event is approximately 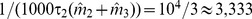 generations, where 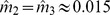 (in Figure 2) is the expected number of single-drug resistant parasites in the migrant pool in a given generation during phase I, if a randomly chosen host is not drug treated at a given time. Since a given host receives repeated drug treatments and drugs do not decay immediately, this expected waiting time is likely an underestimate (see below for more analysis). In host no. 950, the single-drug resistant parasites grow to *N*_C_ and trigger another drug treatment. However, as it takes longer for drugs to reduce *n*_2_ from *N*_C_ to zero than it takes to reduce *n*_1_ from *N*_C_ to zero, these single-drug resistant parasites are not completely cleared before drug concentrations drop below the level inhibitory to their growth. It is therefore followed by the recrudescence of parasitaemia and repeated cycles of drug treatment. Eventually, when the cumulative parasitaemia exceeds *k*_D_ *N*_C_, this infection is terminated (see Methods) and all parasite numbers in the host are set to zero. Before the termination of this infection, however, a large number of single-drug resistant parasites are transferred to the migrant pool, identified by a sharp increase of *m*_2_ (orange curve), which will in turn initiate the infections by the resistant parasites in other, infection free hosts. Figure 2 also shows that *n*_4*j*_ and *m*_4_, the numbers of double-drug resistant parasites (*A_R_B_R_*) in the host and migrant pool respectively, start to increase as *m*_3_ grows to a large number. This causes the recrudescence (relapse of parasitaemia) mostly composed of these double-drug resistant mutants (as happened in host no. 422), which is now impossible to eliminate by drugs, and new infections of double-mutant parasites to other disease-free hosts occur. In this way the double-drug resistant parasites quickly spreads to all other hosts in the population. Overall, Figure 2 shows an example in which the establishment of single-drug resistant parasites in a host is quickly followed by the eventual spread of double-drug resistant mutants in the entire population, thus producing a much shorter phase II relative to phase I. Such a rapid progression was possible as the given intensity of drug treatment (given by *l*_T_ and λ) permitted the recrudescence of single- and double-drug resistant parasites while it prevented the recrudescence of sensitive ones.

**Figure 4:**
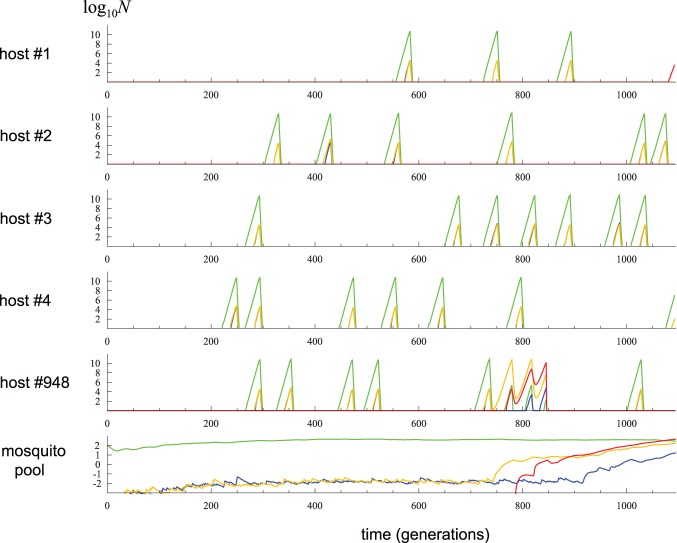
A simulation replicate in which resistance evolution occurs through type B establishment. Parameters were given in Table 1 except for λ = 0.2. See Figure 2 legend for explanations of curves.

In the example shown in Figure 2, the establishment of single-drug resistant mutants (*i.e.* a host becoming predominantly infected by these mutant parasites) occurred due to the transmission of a mutant parasite into an uninfected host carrying no parasites. Such an event is denoted as “type A establishment”. This type of establishment occurred in the majority of simulation replicates that were run using parameters given in Table 1. However, in other replicates, the first establishment resulted from a rare event of recrudescence: an intermediate peak of *n*_2_ or *n*_3_ is reduced by drugs but does not hit zero (*i.e.*, mutant parasites survive) by chance (denoted “type B establishment”). This may happen if a rare early mutation of sensitive to resistant allele, shortly after the inoculation of a sensitive parasite, leads to an unusually large number of single-drug resistant mutants at the time of drug treatment. Obviously, weaker drug treatment (smaller *l*_T_ or larger λ) will promote such an establishment of single-drug resistant parasites through incomplete clearance. Figure 4 shows a particular replicate of simulation (but with λ = 0.2) in which such incomplete clearance happened. When 1,000 replicates were run with parameters given in Table 1, the waiting time until the establishment (either type A or B) had a mean value (±2 s.e.) of 2,490 (±141) generations, and its distribution had an exponential-like shape (although not exponentially distributed). It is much shorter than 3,333 generations, which was expected above assuming that only type A establishments occur.

These exemplary trajectories provide the following insights on the major factors in the model that determine how fast drug-resistant haplotypes spreads in the population: 1) the emergence of drug resistant critically depends on a rare event of resistant mutants’ transmission from drug-treated hosts, through the migrant pool in mosquitoes, to another susceptible host with no or reduced drug concentration (type A establishment), or rare failures of drugs in eliminating mutant parasites (type B establishment); 2) the likelihood of both types of establishments increases as the numbers of single-drug resistant parasites at the time of drug treatment increases; 3) these transient peaks of mutant parasites are determined by the threshold parasitaemia (*N*_C_), the mutation rate (µ) and the selective disadvantages of resistant mutations in the absence of drug pressure (*c*); 4) the rate of type A establishment should increase with the mosquito-to-host transmission rate (τ_2_) and the equilibrium frequency of single-drug resistant mutants in the migrant pool (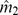 and 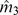) during phase I, which in turn depends on the host-to-mosquito transmission rate (τ_1_) and the relative rates of sensitive and resistant parasites’ elimination by drugs (*d_S_* vs *d_R_*); 5) the rate of type B establishment depends on the intensity of drug treatment (*l*_T_ and λ) for a given *d_R_*; and, 6) once a single-drug resistant haplotype prevails in a host, the emergence of double-drug resistant haplotype quickly follows, provided that the combination drug treatment cannot reduce the number of single-drug resistant parasites from *N_C_* to zero.

### Determinants of waiting times until the establishment of resistance

The model is further examined by simulating drug-resistance evolution with changing parameter values. As the first step in the exploration of parameter space, the value of only one parameter was varied while other parameter values were kept being the “starting values” in Table 1. In this way, the effects of individual parameters were examined. The waiting time (number of generations) until the frequency of the double-drug resistant haplotype (*A_R_B_R_*) in the migrant pool exceeds 0.5 was recorded. The total waiting time, *L*_T_, is divided into the length of phase I, *L*_1_, and phase II, *L*_2_ = *L*_T_ - *L*_1_, where phase I and II are defined above. For each parameter set the means of *L*_1_ and *L*_T_ were obtained over 400 replicates unless stated otherwise. Furthermore, the number of replicates in which the drug resistance spread through type A or type B establishments of single-drug resistant parasites was also counted.

#### Asymmetry of drug strength or monotherapy

First, the impact of asymmetric drug effects (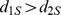 and 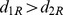) was examined (Table 2). Drug combinations were adjusted such that 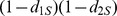 and 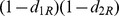, combined drug effects on fully sensitive and resistant parasites, respectively, remain constant (cases 1, 2 and 3 in Table 2). As drugs become more asymmetric, the waiting times (*L*_T_ and *L*_1_) decrease, as the asymmetry accelerates the decay of drug effect (Text S1). When 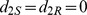, it effectively simulates monotherapy (cases 4–6 in Table 2). To make the result comparable to combination therapy, the effect of the single drug (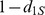) was set to match the effect of combined drugs (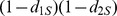) used above and, similarly, 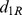 was increased (in case 4 in Table 2, 1–0.9975 = (1–0.95)^2^ and 1–0.84 = (1–0.6)^2^). However, the waiting time was greatly reduced (case 4), firmly supporting the advantage of combination therapy over monotherapy that were claimed in previous studies [1], [23], [33], [41]. Under the framework of the current model there are two main explanations: first, the drug effect decays faster under monotherapy (Text S1); second, a single mutation confers a much higher gain of parasite fitness under monotherapy than under combination therapy. In agreement with the latter argument, an increase in 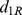 (0.98 in case 5 from 0.84 in case 4 in Table 2), thus stronger drug pressure against the resistant parasites, resulted in waiting times comparable to the cases of combination therapy (case 1–3). However, increasing 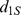 to the extreme value (from 0.9975 to 0.999; case 6) did not have an effect on the waiting time. These results support the conclusion obtained above that the initiation of spread critically depends on the escape of a mutant parasite while their relative frequency within the host increases immediately after drug administration. In the following, only the cases of symmetric combination therapy will be considered, denoting 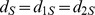 and 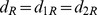 for simplicity.

**Table 2:** Effects of asymmetric drug effects on waiting times (mean ± 2 std. err.).

case	*d*_1*S*_	*d*_2*S*_	*d*_1*R*_	*d*_2*R*_	*L*_T_	*L*_1_
1	0.95	0.95	0.6	0.6	3,074±242.2	2,535±223.8
2	0.975	0.9	0.8	0.2	2,221±167.3	1,830±163.5
3	0.98	0.875	0.84	0	1,808±127.3	1,428±121.8
4*	0.9975	0	0.84	0	120±1.02	14.0±0.48
5*	0.9975	0	0.98	0	2,231±159.8	1,809±159.1
6*	0.999	0	0.84	0	117±1.01	14.3±0.47

*Waiting time until *A_R_* allele reaches frequency 0.5 in the mosquito pool.

#### Mutation rate, treatment threshold, and metabolic cost

**Figure 5:**
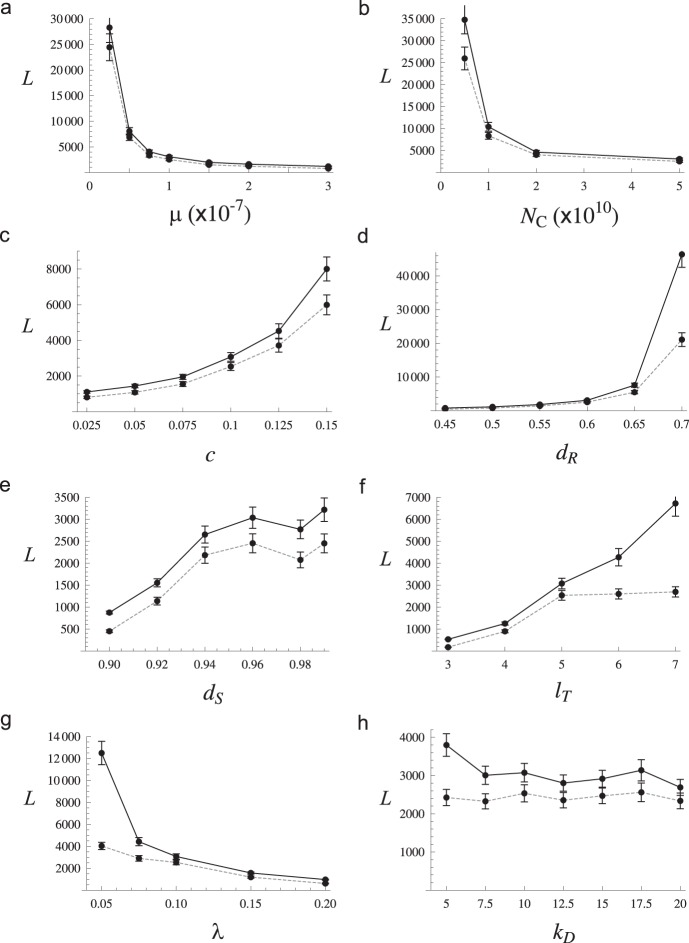
Waiting times until the spread of resistance when a single parameter is varied while others are fixed with values in **Table 1**. The total waiting times (*L*_T_) are shown by points (mean ± 2 standard error) connected by solid lines and the lengths of phase I (*L*_1_) by dashed lines.

Figure 5a shows the negative correlation between µ and *L*_T_ (or *L*_1_): high mutation rate makes drug resistance evolve faster, as expected [16]. The total waiting time is mostly the length of phase I. The threshold parasitaemia for drug treatment, *N*_C_, is negatively related to the waiting time (Figure 5b), indicating that it takes much longer for drug resistance to spread as drug treatments are initiated in response to smaller parasitaemia. The waiting time also increases as metabolic costs (selective disadvantages of a resistant parasites in the absence of drug pressure), *c*, increase (Figure 5c), in agreement with previous studies [21], [23]. These results confirm that the number of single-drug resistant parasites at the time of drug administration, which is modulated by µ, *N*_C_, and *c*, is the critical determinant of the length of phase I through both type A and B establishments: it translates into the expected number of mutants in the mosquito pool (type A) and the probability of failing to eliminate them (type B). It is also shown that *N*_C_ and *c* are negatively and positively related to *L*_2_, respectively. Small *N*_C_ limits the number of mutant parasites transmitted from host to migrant pool during phase II. This result suggests that active surveillance to identify malaria patients and start treatment with a combination drug therapy (or even to treat individuals who are infected but asymptomatic yet) might be a major determinant to delay the initiation and successive spread of drug resistance.

#### Drug efficiency against resistant and sensitive types

With *d_S_* fixed at 0.95, increasing *d_R_* from 0.45 to 0.7 (decreasing the fitness of resistant parasites under maximal drug concentration) results in great increase in *L*_1_ and *L*_2_ (Figure 5d). This is expected as the relative advantage of resistant over sensitive parasites is decreased. In other words, strongly resistant parasites establish and spread quicker than weakly resistant ones, a result that is consistent with the predictions made by other approaches [22]. While increasing *d_R_* gradually increases the length of phase I (*L*_1_), it sharply increases phase II (*L*_2_) at the highest value (0.7). It suggests that, with large *d_R_*, a transient establishment of single-drug resistant parasites in a host does not immediately lead to the spread of resistance to other hosts, because mutant parasites cannot rebound after recurrent treatment. Importantly, this consequently limits the number of resistant parasites transmitted to the migrant pool. The similar (but weaker) effect of *c* on *L*_2_ discussed above (Figure 5c) can be understood by the decreasing relative fitness of resistant types with increasing *c*.

The value of *d_S_* was also changed from 0.9 to 0.99 while *d_R_* was fixed at 0.6 (Figure 5e). Interestingly, increasing *d_S_* (decreasing the fitness of sensitive alleles relative to resistant ones) did not lead to faster spread of drug resistance. *L*_1_ and *d_S_* are positively correlated when *d_S_* increases from 0.9 to 0.94. Then, with *d_S_*>0.94, waiting times are only slightly affected by *d_S_*. This result is explained by the fact that the absolute fitness of a single-drug resistant parasite is a decreasing function of *d_S_* (e.g. 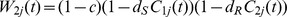). If *d_S_* is sufficiently reduced, the fitness of a single-drug resistant haplotype can increase sufficiently high to allow its rebound after drug treatment, thus initiating the type B establishment of the mutant parasites in a host. In agreement with this explanation, the counts of type B events (out of 400 replicates) were 346, 244 and 100 for *d_S_* = 0.9, 0.92 and 0.94, respectively. Moreover, if *d_S_* is large, the number of parasites within the mosquito pool becomes smaller. Hence, hosts are less likely to be infected (low transmission) than expected due to treatment-induced eradication. Therefore, within a given time window, the number of infections is lower, which implies a longer waiting time for the initiation and subsequent spread of resistance.

#### Duration of treatment and drug decay

Next, the duration (in generation) of the maximum drug concentration after the start of treatment (*l*_T_) was varied from 3 to 7. The complete clearance of both resistant and sensitive parasites during phase I requires a large value of *l*_T_. Therefore, *L*_1_ is expected to increase as *l*_T_ increases, by reducing the likelihood of the type B establishment. Figure 5f shows that this actually happened in the simulation when *l*_T_ increased from 3 to 5. However, *L*_1_ did not change when *l*_T_ increased beyond 5, indicating that *l*_T_ = 5 already reduced the probability of type B establishment to the minimum. When *l*_T_ increased from 5 to 7, *L*_2_ greatly increased. This is because, with a large value of *l*_T_, the number of single-drug resistant parasites is likely to go to zero even when it starts from *N*_C_, thus preventing the spread of resistant haplotypes after a type A (or B) establishment in a host happened.

Similar effects on waiting times were observed when the rate of drug-concentration decay (λ) was varied (Figure 5g). λ = 0.1 means approximately 10% reduction in drug concentration per generation (eq. 9). Smaller λ thus means slower decay, which leads to more thorough elimination of both sensitive and resistant parasites per treatment. Conversely, increasing λ has a similar effect of decreasing *l*_T_. Note that the values of λ considered here are sufficiently large so that, when a parasite is transmitted to a host from the mosquito pool, the host is expected to contain effectively zero concentration of drugs. We observed a negative correlation between λ and the waiting time (i.e. faster evolution of drug resistance with faster decay of drugs; Figure5g), which is contradictory to the current consensus on the expected role of drug decay in drug-resistance evolution [30], [42]. This result will be further discussed below. Additionally, we performed simulation with unequal rates of drug decay: the first drug decays slowly (λ_1_ = 0.05) and the second drug decays fast (λ_2_ = 0.2). In this case, the mean waiting time to resistance (*L*_T_ = 2,836) is much closer to the case of both drugs decaying fast (λ_1_ = λ_2_ = 0.2; *L*_T_ = 971) than slow (λ_1_ = λ_2_ = 0.05; *L*_T_ = 12,498). Therefore, it appears that a drug with faster decay results in rapid emergence of resistance despite the presence of the other slowly-decaying drug.

#### Threshold for host death and recombination rate

The threshold of cumulative parasitaemia causing host death or gravity that justified the treatment by an alternative drug was varied from 5 to 20 times *N*_C_ (Figure 5h). It had little effect on waiting times when *k*_D_≥7.5. However, *L*_2_ increased significantly when *k*_D_ was reduced to 5. This suggests that a very short duration of high parasitaemia inhibits the spread of single (or double)-drug resistant parasites after they are established in a host. This is intuitively clear, because large *k*_D_ implies that resistant parasites can be transmitted efficiently to the mosquito pool. If resistant parasites would be cleared out of a host at the first rebound of parasitaemia (reflecting use of an alternative drug, because of clinical failure), phase II would substantially delay. When additional simulation with *k*_D_ = 50 or 100 was performed, little change in *L*_1_ or *L*_2_ was observed. Therefore, allowing prolonged infection with resistant parasites (due to recrudescence without clinical manifestation) does not seem to speed up the spread of resistance.

Next, the recombination rate *r* was changed from 0.5 to 0 (complete linkage). It caused very little change, from 2,535±223 (mean ± 2s.e.) to 2,508±211 for *L*_1_ and from 3,074±242 (mean ± 2s.e.) to 3,094±235 for *L*_1_+ *L*_2_. This result is in contrast to earlier studies that predicted the profound effect of recombination in slowing down the resistance evolution under combination therapy [1], [20] (see Discussion).

#### Proportion of infected hosts that are treated

So far, we considered scenarios in which all infected hosts are detected and treated. Now, only proportion α of hosts receive drug treatment because carrying capacity for parasites (*K* in eq. 1) in other hosts is set below *N_C_*. Decreasing α increased both *L*_1_ and *L*_2_: with α = 0.9, 0.8, 0.7, 0.6, and 0.5, *L*_T_ (*L*_1_) = 2,707 (2,123), 2,930 (2,222), 3,796 (2,788), 4,802 (3,332), and 6,283 (4,253), respectively. Waiting times could not be obtained with α<0.5 as it prevented resistant alleles from reaching relative frequency of 0.5 in the migrant pool. This result confirms the conclusion of previous studies that the presence of untreated hosts confers advantage to sensitive parasites and thus delay the spread of drug resistance [16], [22].

#### Effects of transmission rate and pattern

**Figure 6:**
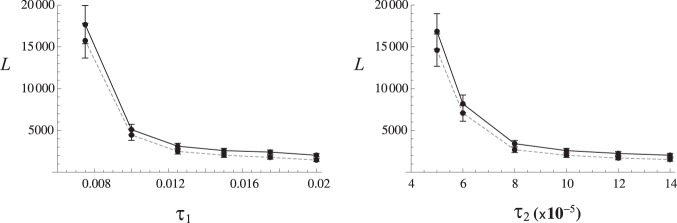
Waiting times until the spread of resistance when host-to-mosquito (τ_1_) and mosquito-to-host (τ_2_) transmission rates are varied while other parameters are fixed with values in Table 1 (except *k*_D_ = 50 and α = 0.9). The total waiting times (*L*_T_) are shown by points (mean ± 2 standard error) connected by solid lines and the lengths of phase I (*L*_1_) by dashed lines. Low transmission rates (τ_1_<0.007 and τ_2_<5×10^−5^) caused the complete elimination of parasites in total population upon the start of drug treatment, which prevented further simulation. Mean times to waiting times are based on 200 replicates.

The population structure of parasites in the current model, over which resistance spreads as described above, is very sensitive to transmission rate τ_1_ and τ_2_. If fewer parasites migrate to the mosquito pool, it leads to fewer hosts infected, which further reduces parasite numbers in mosquito pool. Therefore, as τ_1_ or τ_2_ decreases gradually to certain thresholds, the overall population size of parasites declines drastically, eventually leading to population collapse (*i.e.* drug treatments leading to complete clearance of parasites in the entire system). It is reflected in very rapid increase of *L*_T_ as τ_1_ or τ_2_ becomes smaller than the values used above (Figure 6): as population size becomes smaller mutations arise less frequently to initiate resistance evolution. Such high degree of sensitivity to transmission rates might be due to the unrealistically small number of hosts used in simulation. However, the general negative relationship between transmission rates and the waiting time is likely to hold in actual (larger) population. (Note – This result should be understood separately from many studies’ conclusion that resistance occurs more rapidly in low transmission areas, which is based on higher fraction (α) of hosts being treated upon infection in lower transmission areas. Our result was obtained while α is kept constant.).

We also examined if the unique conclusion from our model, in particular the positive relationship between λ and *L*_T_, depends on the assumption in eq. (4) that the host-to-mosquito transmission rate is proportional to 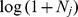. Simulation was performed after this term was replaced by 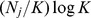, so that the transmission rate linearly increases with parasitaemia until it reaches *K*. Without increasing the transmission rate, this change led to the collapse of parasite population: during the early phase of infection when *N*<<*K* too few parasites migrate into the mosquito pool to sustain the cycle of infections while each infection reaching *N*_C_ is knocked down by drug treatment. τ_1_ was thus increased to 0.05 (from 0.015). Under this setting, while the average number of parasites maintained in the migrant pool was similar (approximately 300), the expected numbers of single-drug resistant parasites (*m*_2_ and *m*_3_) were more than an order magnitude smaller (*m*_2_/*m*_1_ ∼10^−6^; it was ∼10^−5^ in Figure 2 and 4). It therefore led to longer waiting time for the establishment of resistance. This demonstrates that the effect of non-linear function (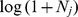) in host-to-mosquito transmission rate to confer transmission advantage to resistant parasites, as explained earlier (Figure 3). The relationship between λ and *L*_T_ was still negative (for λ = 0.075, 0.1, 0.15, and 0.2, *L*_T_ = 34,076, 8,599, 1,134, and 625 generations, respectively), suggesting that the qualitative behavior of the system did not change. In addition, we considered a scenario in which transmission rate (frequency of mosquito bites) is higher for certain hosts than others [43]. Simulations were performed with host-to-mosquito transmission according to eq. (4) and with parameter values in Table 1 except that different values of τ_1_, uniformly distributed between 0 and 0.03, were assigned to individual hosts. Transmission rate to a given host (τ_2_) was also changed proportionally (τ_2_ = τ_1_/1500). Such heterogeneity in host-to-mosquito transmission however did not change the overall behavior of dynamics and the relationship between λ and *L*_T_ (for λ = 0.05, 0.075, 0.1, 0.15, and 0.2, *L*_T_ = 12877, 4850, 3046, 1632, and 960 generations, respectively).

## Discussion

This study proposed a model of the evolution of anti-malarial drug resistance which focuses on the early stochastic processes including *de novo* mutations conferring partial resistance against combination therapy and their successful propagation under weak intra-host competition. Our model was designed to investigate the joint dynamics of both parasite numbers and haplotype frequencies by tracking the absolute as well as relative counts of sensitive vs. resistant parasites. Such an approach was particularly necessary to address the notion that the complete elimination of parasites by strong drug treatment may effectively prevent the evolution of resistance [31].

### Pathway to the first establishment of resistance mutation

The occurrence and initial establishment of resistant parasites is a major determinant of the timeframe of drug resistance evolution [26], [44]. The initial propagation of resistant alleles, which emerges within an infection by random mutation, can continue only if it is transferred to a mosquito. However, if the initial mutation only partially restore parasites’ fitness under drug treatment (e.g. tolerance as have been currently observed in ACTs), drugs will likely eliminate resistance parasites before they can enter the transmission cycle and, therefore, the spread of resistance cannot be explained. To circumvent this problem, the potential role of sub-optimal drug concentration, at which sensitive parasites are killed (absolute fitness <1) but resistant parasites are not (absolute fitness >1), during the period of drug decay was invoked [30], [42]: a parasite carrying a resistant allele may enter a host with suboptimal drug concentration and multiply to establish its clone. This will initiate the cycles of infection to other hosts (also with suboptimal drug concentration). This model can thus explain the spread of initially weakly resistant alleles [30], [42]. However, the very first appearance of a resistant parasite to be transmitted to a host remained unexplained, and thus implicitly assumed that they somehow arose in a mosquito vector. It might be possible that a *de novo* mutation occurs during meiosis within the mosquito gut [25]. However, this scenario might be ignored because significantly lower numbers of parasites are carried within mosquitoes relative to those within hosts [44]. Recently, Pongtavornpinyo *et al.* [26] assessed the probability of the initial spread of *de novo* mutation in different stages of parasite’s life cycle. However, this study assumed mutations conferring full resistance and the probability of resistant parasites surviving treatment - the key determinant of the model - was externally given rather than given as a function of more basal clinical and pharmaceutical parameters. In the current study, the described intrahost dynamics is much simpler. However, as we made minimal assumptions regarding the fate of resistant alleles, computer simulations of our model revealed key events leading to the onset and spread of resistance.

### Incomplete resistance by initial mutation and absolute fitness

A critical assumption of this model is that mutation at each locus confers only partial or incomplete resistance: the absolute fitness of resistant parasite is below one under the full dose of the corresponding drug. In addition, this absolute fitness is only slightly dependent on the parasitaemia of the host (cf. “specific immunity” in [21]). Namely, when sensitive parasites are eliminated by drugs, the fitness of resistant parasites in the same host is not sufficiently increased to yield their positive growth. This is in contrast to the “competition” scenario in which a strong density regulation (presumably due to resource competition) is assumed to cause the increase of resistant parasites in place of killed sensitive parasites (“generalized immunity” in [21]; also [33], [34]). The competition scenario implicitly assumes that total parasitaemia in a host is always maintained above zero despite drug treatment. Namely, the “extinction” of local population does not occur. Then, the evolutionary of trajectory of parasites is fully determined by the *relative* fitness between sensitive and resistant parasites. Since the mutants’ relative fitness only increases as stronger and longer-lasting drugs are used, the competition scenario inevitably leads to a conclusion that resistance spreads faster with more aggressive drug treatment. In contrast, in our model, the higher relative fitness of resistant over sensitive haplotypes does not lead to an immediate increase of resistant parasites because the *absolute* fitness of resistant parasites under treatment, free from competition effect, is still below one. Furthermore resistant parasites are more quickly removed before they enter transmission cycle by stronger drugs. This leads to the opposite conclusion that aggressive drug treatment delays the evolution drug resistance.

### Delayed resistance with aggressive treatment and slow drug decay

The simulation results indicate that the spread of drug resistance is delayed when the drug treatment is strong (large values of *d_R_* and *d_S_*) and long-lasting (large *l*_T_ and small λ), which ensures the complete elimination of not only resistant parasites that accumulate to a modest number by mutations but also sensitive parasites. Using drugs with strong effect on sensitive parasites (large *d_S_*) is important as it reduces the opportunities for type A (by reducing the source of mutants transmitted to the mosquito pool) and type B (by reducing the fitness of single-drug resistant parasites that are being eliminated) establishments. It is also suggested that an early detection of infections and drug use, which lowers the parasitaemia (*N_C_*) at the time of treatment, can delay the evolution of resistance. These results point to the general population-genetics principle that the rate of adaptive evolution is proportional to the size of population from which rare variants arise: with a smaller number of sensitive parasites maintained under aggressive drug treatment, a smaller number of resistant parasites will be produced by mutation. With reduced production of resistant parasites, the opportunity of their transfer to mosquito vector and subsequent propagation to other hosts becomes severely limited.

It is of particular note that a slow decay of drug concentration (decrease in λ in eq. 9) delays the evolution of drug resistance (increase in waiting time) given that other parameter values are held constant. Other studies have offered the opposite conclusion that a slow decay promotes the evolution of resistance [30], [34], [42]. According to these studies, a slow decay of drugs extends the time window of suboptimal drug concentration at which the growth rate of resistant parasites is positive while that of sensitive ones is negative. However, we do not find such predicted effect of extended suboptimal drug concentration in accelerating resistance evolution. This discrepancy between our result and their prediction is again due to our model based on absolute fitness of parasites that makes it possible to eliminate partially resistant mutants with drug treatment. Here, λ is not a simple parameter that specifies the rate of drug decay only: with other parameters fixed, a decrease in λ implies an increase of drug efficiency due to increase in cumulative drug dose. When initial partially resistant mutants are efficiently eliminated, the emergence of resistance would be greatly delayed. It should also be noted that, in the simulation results, after a partially resistant allele’s initial establishment (type A or B) in a host, its spread to other hosts does not depend on the presence of a suboptimal drug concentration: it is mainly transmitted to parasite-free hosts with zero concentration of drugs and then quickly multiplies to a large number without having to compete with sensitive parasites. Therefore, the importance of suboptimal drug concentrations in promoting resistance evolution, as advocated in previous studies, may not apply to our model. However, we admit that, due to the simplicity of intrahost reproduction in our model, the role of suboptimal drug concentration in enriching resistant parasites after they exit the hepatic stage of their life cycle [26] could not be addressed. Further refinement of our model by dividing a host into hepatic and blood stage will be needed for accurate assessment of this problem.

### Limits in the model

Although the model studied here provided important insights on the complex stochastic processes involving the initial spread of partially resistant mutations, how accurately it represents the actual pathway of resistant parasites’ spread might be debatable. First, due to the lack of detailed information regarding the realistic ranges of many model parameters and the constraint in the number of hosts simulated, the behaviour of the model was examined for arbitrary sets of parameters. We did not observe any indication that the qualitative dynamics of the system depends on any particular range of parameter values, as the waiting times *L*_1_ and *L*_T_ increased or decreased largely monotonically as predicted when the value of a single parameter changed (Figure 5). However, it is possible that joint changes in several variables toward (unknown) realistic values may lead to a qualitatively different behaviour of the system.

Second, the current model of intrahost dynamics might be too simplistic to reveal important factors of resistance evolution including the effect of recombination. No effect of recombination was observed in our simulation probably because, in the glimpse of Figure 2, haplotypes 1 and 4, or 2 and 3, rarely coexist in sufficient frequencies in a host. This non-overlap between haplotypes that preclude recombination is expected under the current model of simple within-host parameter growth: unless two different strains enter a host at very similar time points, their numbers are expected to be highly unequal because of the rapid exponential growth of the first clone before the arrival of the second. Given that multiple clones of *Plasmodium* are frequently detected from a single host [31] and therefore provide the evidence that frequent recombination between different parasites actually occurs [5], the model of within-host parasite growth needs to be modified in the future to allow similar parasitaemia for different clones.

More importantly, our model of intrahost parasite reproduction imposed only very weak competition between strains of different haplotypes. Using a rodent malaria model, Wargo *et al*. [35] experimentally demonstrated that the removal of sensitive parasites by drug treatment led to an amplified release of resistant parasites after treatment. It is worth noting that there are differences between the rodent malaria model and *P. falciparum* that may affect the generalization of these results [35]. However, if such strong intrahost competition exists in human malarias, the overall dynamics of resistance evolution may drastically change such that aggressive drug treatment facilitate the spread of resistant parasites [33], [34]. It is not clear how a mathematical model can be built for such strong competitive exclusion between strains. We explored several ways of modifying our reproductive model to impose strong competition. For example, we replaced the density-regulatory factor 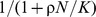 in eq. (1) by 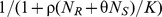 for the absolute fitness of a (single-drug) resistant parasite where *N_R_* and *N_S_* are the numbers of resistant and sensitive parasites and θ is the competition factor. Using θ>>1 and changing other parameters to increase the resistant mutant’s fitness one can model the rapid growth of resistant parasites when the host is “free” of sensitive parasites. However, this condition also prohibits the accumulation of resistant parasites by spontaneous mutations while sensitive parasites prevail before treatment, leading to unrealistically long waiting times (data not shown). Thus it appears that a new mathematical model beyond simple density regulation of parasites needs to be developed to investigate the effect of intrahost competition on drug-resistance evolution in realistic time scale.

Finally, a profound understanding of the evolutionary mechanisms driving drug resistance - from initial establishment to successive progression - under combination therapy is most important, as combination therapy was argued to be the most promising strategy [41]. Our model provides such insights into drug resistance, while emphasising the importance of the two phases involved in establishing drug resistance. While the present study clearly showed that combination therapy is superior to monotherapy, testing combination therapy against alternative strategies (alternating therapy, mosaic therapy cf. [41]) requires further investigation. Our model can be easily adapted for this purpose.

## Supporting Information

Text S1 **Effectively faster decay of drugs under monotherapy.**
(DOC)
