# Supplementary material for: A Population Genetic Model for the Initial Spread of Partially Resistant Malaria Parasites under Anti-Malarial Combination Therapy and Weak Intrahost Competition
Source: PLoS One. 2014 Jul 9;9(7):e101601. doi: 10.1371/journal.pone.0101601 (PMC4090191; doi:10.1371/journal.pone.0101601)
Supplement: Text S1 — Effectively faster decay of drugs under monotherapy. (DOC) [file pone.0101601.s001.doc]

Supporting Text S1. Effectively faster decay of drugs under monotherapy

Let $d_{S}$ and $d_{R}$ be drug effects in the combination therapy and $\tilde{d}_{S}$ and $\tilde{d}_{R}$ be those for monotherapy. If subscripts are skipped for simplicity,

$(1-d)^{2}=1-\tilde{d}$.

Hence, $\tilde{d}=2d-d^{2}$, which implies for λ > 0 and *t* > 0

$\tilde{d}<2d-d^{2}e^{-\text{λ}t}$.

When both sides of the inequality are multiplied by $e^{-\text{λ}t}$, after rearrangement

$(1-de^{-\text{λ}t})^{2}<1-\tilde{d}e^{-\text{λ}t}$.

Therefore, even if the parasite fitness are equal for both treatment options under maximum drug concentration, the absolute fitness is restored faster under monotherapy than under combination therapy as drug concentration decays. The same reasoning applies to asymmetric combination therapy.
